# Impact of aging on meningeal gene expression

**DOI:** 10.1186/s12987-023-00412-9

**Published:** 2023-02-06

**Authors:** Melanie Neutzner, Corina Kohler, Stephan Frank, Hanspeter E. Killer, Albert Neutzner

**Affiliations:** 1 Department of Biomedicine, University Hospital Basel, University of Basel, Hebelstrasse 20, 4031 Basel, Switzerland; 2 Department of Pathology, University Hospital Basel, University of Basel, Basel, Switzerland

**Keywords:** Meninges, Aging, Cerebrospinal fluid, Neuroimmunology, Neurodegeneration, Inflammation, Brain

## Abstract

**Background:**

The three-layered meninges cover and protect the central nervous system and form the interface between cerebrospinal fluid and the brain. They are host to a lymphatic system essential for maintaining fluid dynamics inside the cerebrospinal fluid-filled subarachnoid space and across the brain parenchyma via their connection to glymphatic structures. Meningeal fibroblasts lining and traversing the subarachnoid space have direct impact on the composition of the cerebrospinal fluid through endocytotic uptake as well as extensive protein secretion. In addition, the meninges are an active site for immunological processes and act as gatekeeper for immune cells entering the brain. During aging in mice, lymphatic drainage from the brain is less efficient contributing to neurodegenerative processes. Aging also affects the immunological status of the meninges, with increasing numbers of T cells, changing B cell make-up, and altered macrophage complement.

**Methods:**

We employed RNASeq to measure gene expression and to identify differentially expressed genes in meninges isolated from young and aged mice. Using Ingenuity pathway, GO term, and MeSH analyses, we identified regulatory pathways and cellular functions in meninges affected by aging.

**Results:**

Aging had profound impact on meningeal gene expression. Pathways related to innate as well as adaptive immunity were affected. We found evidence for increasing numbers of T and B lymphocytes and altered activity profiles for macrophages and other myeloid cells. Furthermore, expression of pro-inflammatory cytokine and chemokine genes increased with aging. Similarly, the complement system seemed to be more active in meninges of aged mice. Altered expression of solute carrier genes pointed to age-dependent changes in cerebrospinal fluid composition. In addition, gene expression for secreted proteins showed age-dependent changes, in particular, genes related to extracellular matrix composition and organization were affected.

**Conclusions:**

Aging has profound effects on meningeal gene expression; thereby affecting the multifaceted functions meninges perform to maintain the homeostasis of the central nervous system. Thus, age-dependent neurodegenerative processes and cognitive decline are potentially in part driven by altered meningeal function.

**Supplementary Information:**

The online version contains supplementary material available at 10.1186/s12987-023-00412-9.

## Introduction

Consisting of three distinct layers-dura, arachnoid and pia mater—the meninges cover and protect the brain, the spinal cord and the optic nerve. Embedded between pia and arachnoid mater, the subarachnoid space (SAS) is filled with cerebrospinal fluid (CSF). Aside from physically enveloping the central nervous system (CNS), the meninges harbour stem cell niches [1], provide neurotrophic factors influencing brain development [2, 3], perform immune surveillance of the CNS [4], and are critically important for CSF homeostasis [5, 6].

The meninges are an important interface between the immunologically privileged CNS and the immune system. While pial vessels consist of endothelial cells forming tight cell–cell contacts, fenestrated vessels in the dura allow for the exchange of large molecules and plasma components [7]. In addition, the meninges are host to a lymphatic system [8], which drains CSF to the deep cervical lymph nodes allowing for immunological surveillance of this fluid compartment [9]. Interestingly, there are strong indications that age-dependent dysfunction of meningeal lymphatic drainage is linked to the development of neurodegenerative diseases [10, 11]. Furthermore, the meninges are populated by a host of immune cells including macrophages, dendritic cells, mast cells, neutrophils, and B and T lymphocytes, the latter comprising CD4 + , CD8 + , γδ and regulatory T (T_reg_) subpopulations [12–14]. Whereas age-dependent increases in T_reg_ numbers are linked to age-associated cognitive decline [15], IL-17 secreting γδT cells, which start populating the meninges shortly after birth, are important for behavioural development [16]. Moreover, early in life, meningeal B cells originating from the skull bone marrow form a subset distinct from systemic B cells [17]. During aging, however, systemic B cells migrate into the meninges, thereby changing the meningeal adaptive immunity landscape [17].

Originating from yolk sac precursors early during development, meningeal macrophages (mMΦ) are closely related to parenchymal microglia. These cells can persist over long periods of time and have the potential for extensive self-renewal [18]. Like microglia in the parenchyma, mMΦ are dynamic and constantly survey their surroundings. Various mMΦ subsets can be distinguished based on their marker expression, with pia mater enriched in Lyve-1 and MHC-II positive macrophages, and the dura containing mainly Lyve-1^−^ and MHC-II^+^ macrophages [19].

In addition to the extensive complement of immune cells [20], the meninges also house a large population of fibroblast-like meningothelial cells (MECs). Reflecting the multiple functions of the meninges, these cells represent a transcriptionally diverse spectrum of cell types [21]. Within the dura, outer periosteal dural fibroblasts and inner dural border cells form a barrier towards the cranial bone and the arachnoid, respectively. Immediately adjacent to the inner dural border, arachnoid barrier cells form a tight layer separating the CSF filled subarachnoid space from the dura with its fenestrated vessels. The arachnoid extends across the subarachnoid space with arachnoid fibroblasts either as in the mouse by directly making contact with the pia underneath or by covering collagenous septae and pillars as in humans and rats. Finally, pial fibroblasts delimiting the subarachnoid space opposite of the arachnoid form a basement membrane with direct contact to the brain parenchyma [20, 22]. Along with their different location, these fibroblasts express various cell–cell contact proteins [23] explaining differences in barrier formation on the dural, arachnoid and pial level. One important function of MECs is maintaining the CSF and the microenvironment of the subarachnoid space. MECs are highly active facultative phagocytes capable of ingesting neurotoxic peptides such as Aβ and α-synuclein [24], gram-positive and gram-negative bacteria [25], as well as apoptotic bodies [26]. During inflammation, MECs are also involved in T and B cell recruitment and activation [27]. In addition, and further connecting these cells to the meningeal immune system, MECs react to pyrogenic signals by secreting pro-inflammatory cytokines [20, 28, 29]. Furthermore, MECs are a major source of extracellular matrix components for maintaining meningeal integrity and function [30].

Using RNASeq, we analysed the effects of aging on this complex anatomical structure. We identified aging-induced changes to the meningeal transcriptional profile consistent with a pro-inflammatory phenotype that includes the accumulation of T and B lymphocytes as well as changes in the activation status of mMΦ. Furthermore, our data indicate that aging not only affects the complement system, but also impinges on chemokine and cytokine signalling. Finally, we also observed age-related changes in meningeal transport mechanisms involving solute carriers and endocytosis. As meninges are critically important for maintaining CNS function, these changes might play a role in the context of neurodegenerative processes associated with aging.

## Results

To understand the effect of aging on meninges, we performed transcriptome analysis of this tissue isolated from young (n = 9, 13.2 ± 2.7 weeks) and aged (n = 12, 87.8 ± 5.1 weeks) female C57BL/6JRj mice. Following perfusion of animals to remove blood, meninges were isolated from the calotte [31]. No meninges were dissected from the surface of the brain to minimize sample contamination with neural tissue.

**Fig. 1:**
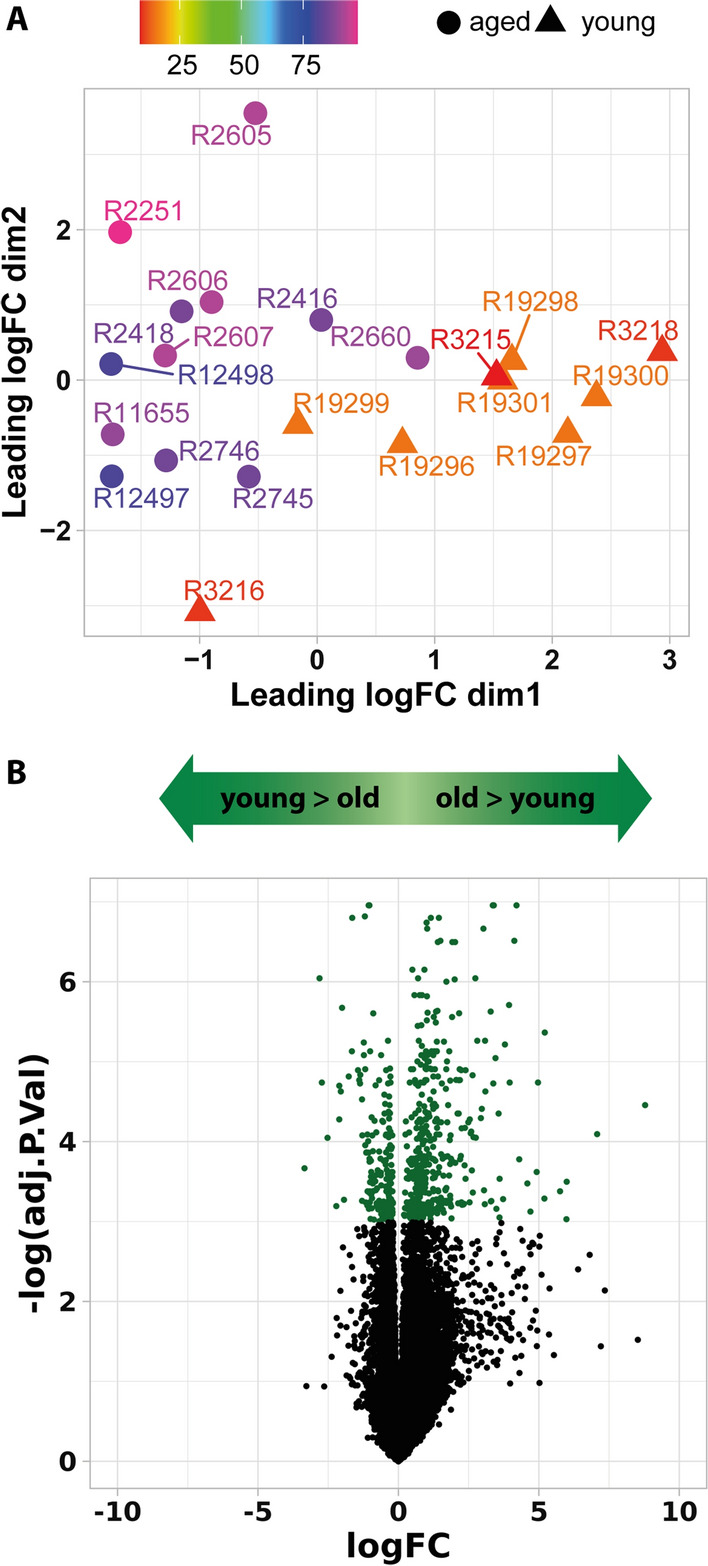
Comparing gene expression in meninges isolated from young and aged mice. **A** Meninges were isolated from mice at different ages, total RNA was isolated, and gene expression was determined by RNASeq. Shown is a multi-dimensional scaling analysis of 21 samples based on the top 250 differentially regulated genes. **B** Volcano-plot of differential gene expression plotted as log_10_ fold change vs. statistical significance of observed differential gene expression expressed as -log_2_ of the Benjamini–Hochberg adjusted p-value

Following total RNA isolation, RNAseq was performed to obtain 300 bp long paired sequences, which were aligned to the mouse reference genome using subread (Table S1). Count data were filtered against lowest level expressed genes (counts per million < 1), normalized using edgeR and prepared for linear modeling using voom ([32]; Additional file 1: Fig. S1). Multidimensional scaling clearly separated between the two age groups (Fig. 1A). Differential gene expression analysis revealed that 177 out of 18240 identified genes were downregulated in aged compared to young animals, while 429 genes were upregulated Fig. 1B and Additional file 1: Fig. S1). To exclude that aging altered the physical properties of meninges to an extend interfering with unbiased sampling, we compared expression of known meningeal cell marker genes [21] between young and aged animals. We found no significant difference in expression of the dural markers *Fxyd5* and *Mgp*, the dural/arachnoid marker *Crapb2*, the arachnoid marker *Aldh1a2* (*Raldh2*) and *Cdh1* (E-cadherin), the arachonoid/pial marker *S100a6*, and the pial marker *Lama2*. Similarly, the lymphatic marker *Lyve1* was not differentially expressed between the age groups (Additional file 1: Fig. S2). Based on these data, aging did not cause bias in our sample preparation and our sample likely contains to some extent fibroblast from all three meningeal layers despite preparing sample only from the calvaria bone.

### Immune-related pathways are affected by aging

**Fig. 2:**
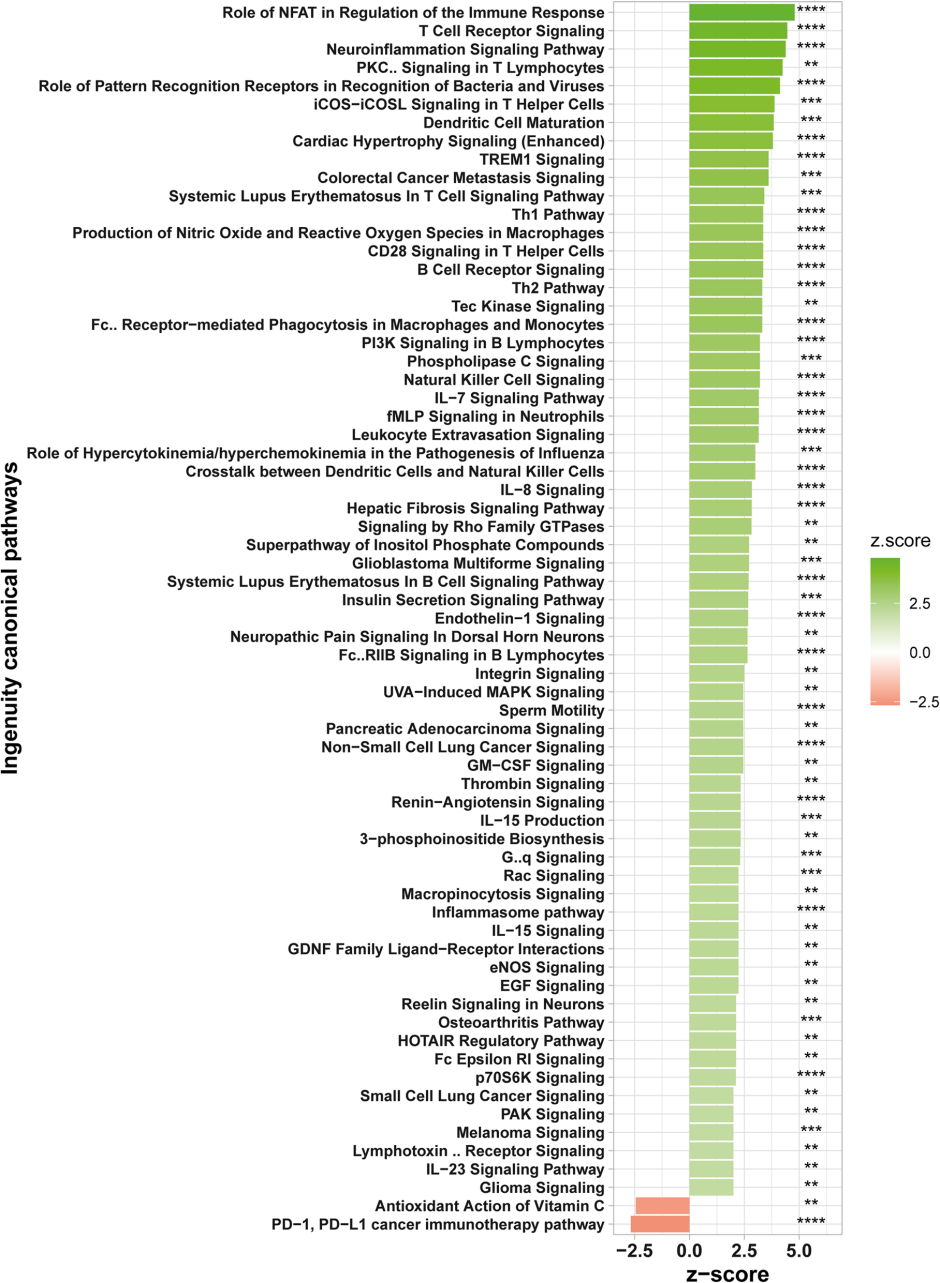
Ingenuity analysis – Canonical pathways. Ingenuity canonical pathway analysis was performed on the young vs. aged meningeal genes data set. The bar graph shows canonical pathways differentially regulated in meninges between young and aged mice. Only pathways with log_10_ Benjamini–Hochberg adjusted p-value > 2 and z-score >|2| are shown (** adjusted p-value < 0.01, *** adjusted p-value < 0.001; **** adjusted p-value < 0.0001)

Using Ingenuity Pathway Analysis (IPA), we found several regulatory networks influenced by the aging process (Fig. 2). This analysis revealed a clear impact of aging on meningeal gene expression related to immune response-related signaling. Among the affected pathways are T and B cell receptor signaling, pathways targeting dendritic cells, natural killer cells, macrophages and monocytes. In addition, aging affects inflammasome and interleukin signaling as well as signaling from pattern recognition receptors.

Next, we performed cluster analysis on the results reported by the IPA to identify genes shared between the identified pathways allowing for a more detailed subanalysis (Additional file 1: Fig. S3). We found *Pik3r2* (93/116 pathways) and *Pik3r5* (89/116 pathways) coding for phosphoinositide-3-kinase regulatory subunits 2 and 5 as well as Pik3Cd (92/116 pathways, coding for phosphatidylinositol-4,5-bisphosphate 3-kinase catalytic subunit δ) which is central to most identified pathways. Phosphoinositide 3-kinases and their regulators are critical immune response signaling molecules, as are members of the integrin family Itgb2 (24/116 pathways), Itgal (20/116 pathways), Itgam (19/116 pathways), and Itgax (18/116 pathways) or members of the MHC class II complex (13-15/116 pathways). Other highly relevant genes include *Relb* (56/116 pathways) coding for an NF-κb regulatory factor and *Plcg2* (51/116 pathways) coding for phospholipase C gamma2 involved in immune receptor signal transduction.

**Fig. 3:**
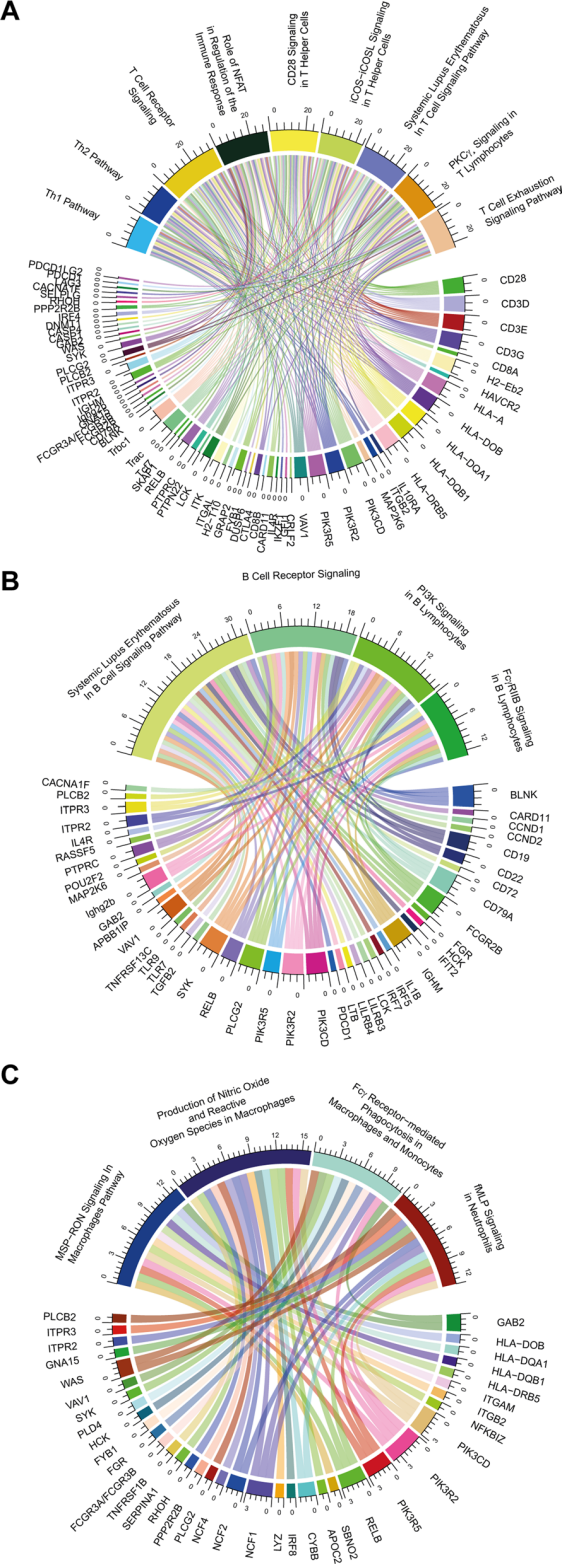
Aging affects multiple pathways involving T and B lymphocytes and myeloid cells. Differentially expressed genes and pathways selected by Ingenuity analysis related to (**A**) T cells, or (**B**) B cells, or (**C**) myeloid cells were plotted in a circos diagram. Please note the involvement of certain genes in multiple pathways

Most prominent and with highest activation score, pathways related to T lymphocytes were affected by aging. Among the regulated genes most commonly connected to these pathways are *Cd3d/e/g* and *Cd28* coding for the T cell receptor and its co-stimulator, *Pik3cd, Pik3r2, Pik3r5* and *Vav1* involved in T cell receptor signal transduction. In addition, genes coding for MHC class II complexes expressed on antigen presenting cells for T cell stimulation are also upregulated (Fig. 3A).

Pathways related to B lymphocytes were less affected by aging. Prominent genes related to these four B cell-related pathways are *Blnk* involved in B cell activation, *Syk* important for B cell receptor signaling and *Relb* critical for B cell maturation and survival (Fig. 3B). Similarly, pathways related to myeloid cells such as macrophages and neutrophils were influenced by aging. Of note here are *Lyz2* coding for lysozyme found in macrophages and granulocytes, and *Ncf1/2/4* coding for neutrophil cytosolic factors (Fig. 3C).

**Fig. 4:**
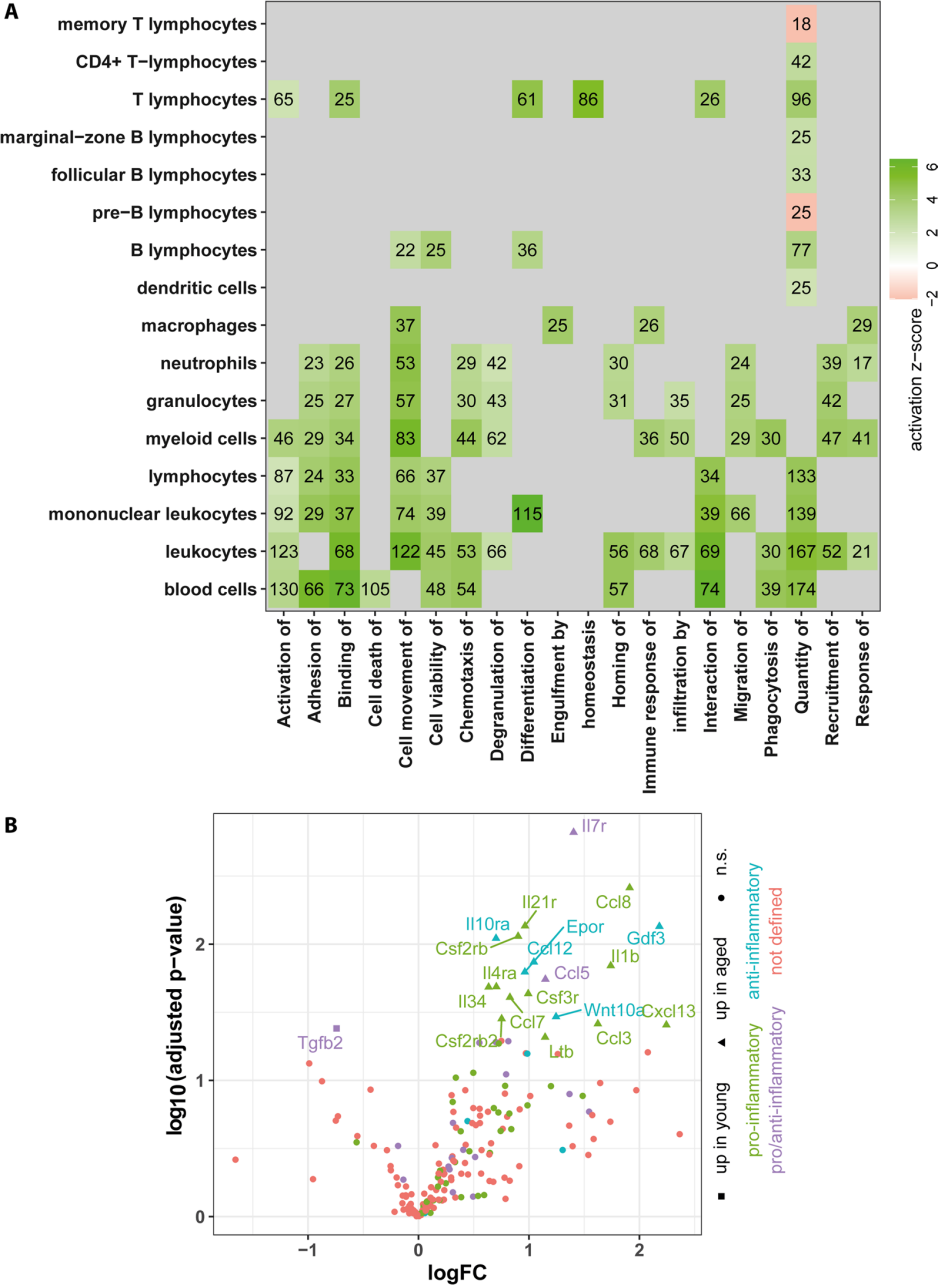
Aging impacts number and activation state of cells of the innate and adaptive immune system. **A** Based on Ingenuity “disease and function” analysis, functions related to cells of the immune system were selected and their activation z-score as well as the number of genes for each affected function were plotted. **B** Using GO term analysis, a list of genes related to cytokines and chemokines was assembled and functionally annotated. Shown are genes differentially expressed between young and aged meninges

Since the analysis of canonical pathways suggested a strong impact of aging on cellular signaling pathways of the innate as well as the adaptive immune system, we also performed an Ingenuity-based diseases and function analysis. Interestingly, when categories of immune cells vs. reported functions were plotted, a clear divide in activation between functions associated with cells of the adaptive immune system versus cells of the innate immune system is evident (Fig. 4A). Of note, while T and B cell functions are clearly influenced, functions related to myeloid cells show more widely ranging activation. When further comparing adaptive and innate immunity, the quantity of B and T lymphocytes but not of myeloid linage cells seemed to be increased. Nevertheless, several functions (such as adhesion, binding, movement, chemotaxis, homing, infiltration, migration and recruitment) seemed to be affected by the aging process in granulocytes and neutrophils. Moreover, functions associated with macrophages such as engulfment and response are also activated, although by a comparably lesser degree.

In line with the age-associated increase in meningeal immunological activity, IPA also suggested increased interleukin and chemokine signaling with aging. To further elucidate this, we generated a gene list of cytokines and chemokines as well as their cognitive receptors using GO term analysis (GO:0008009, GO:0004950, GO:0005125, and GO:0004896 for "chemokine activity", "chemokine receptor activity", "cytokine activity", "cytokine receptor activity"). In addition, we assigned labels for pro- and anti-inflammatory action to this gene list based on associated GO terms. Based on this list of 197 genes, we found 19 genes to be upregulated in aged meninges of which twelve were annotated with pro- and five with anti-inflammatory action, while two genes had both pro- and anti-inflammatory annotation. In young animals, only one of these genes with ambiguous annotation was expressed at higher levels as compared to aged animals (Fig. 4B). Collectively, these data point to an age-dependent increase of pro-inflammatory signaling in the meningeal compartment. Together with the apparent increase in B and T cells, it seems that the meningeal innate and adaptive immune systems enter a heightened activity state during aging.

### Meningeal transport mechanisms and aging

**Fig. 5:**
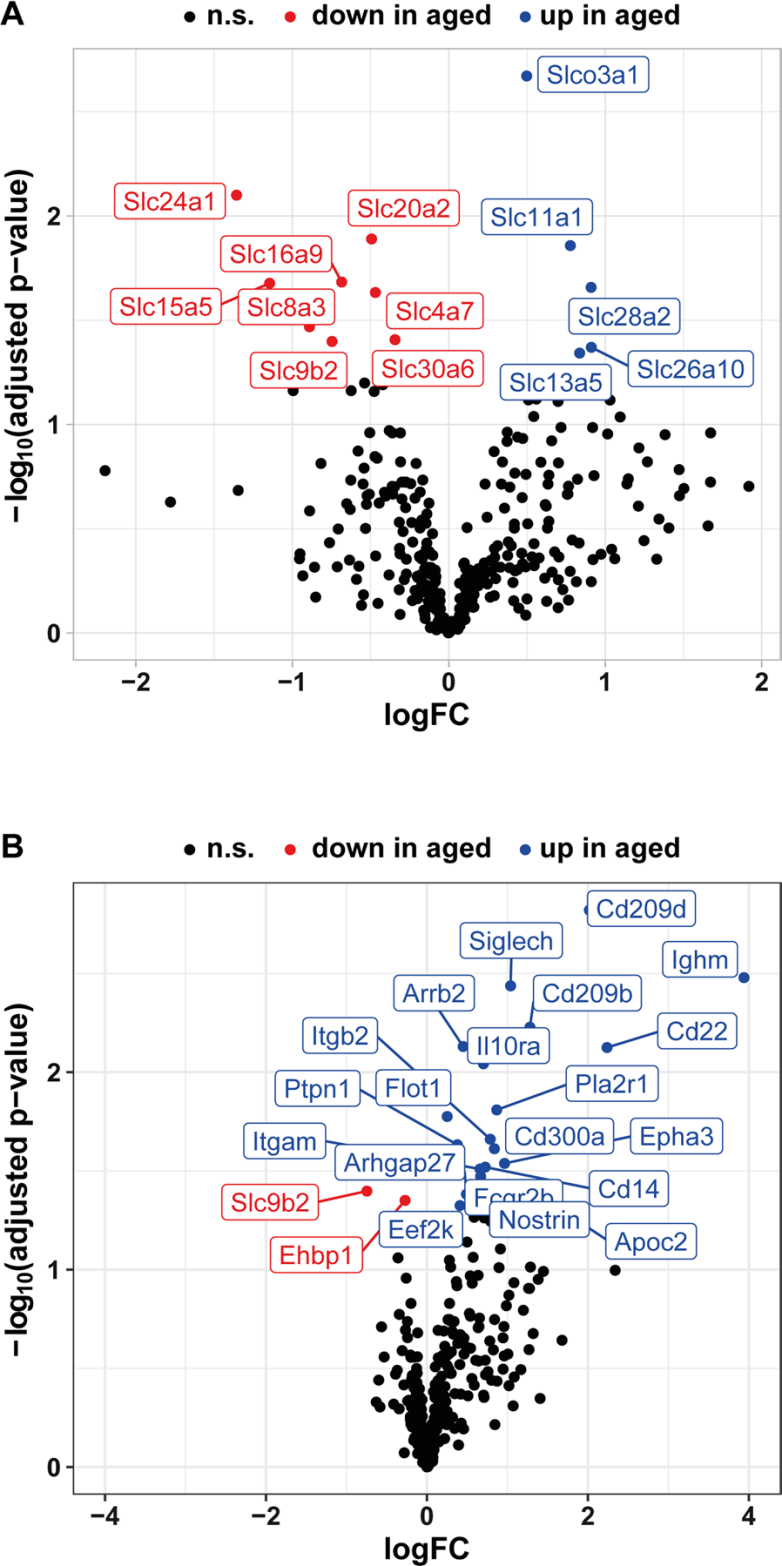
Impact of aging on meningeal transport mechanisms. **A** A gene list containing 334 genes coding for solute carrier proteins expressed in meninges was generated. The volcano plot highlights eight down- and five upregulated solute carrier genes. **B** Using GO term analysis, a list of genes coding for proteins involved in endocytosis was assembled. The volcano plot highlights differentially expressed gene of this group of genes

As part of the blood-CSF barrier, the meninges play important roles in maintaining CSF homeostasis. In addition to meningeal lymphatic vessels draining CSF, direct uptake but also secretion by MECs and resident immune cells contributes to CSF composition. Thus, we first analyzed the class of solute carriers, some of which were previously shown to be active in meninges. Out of 334 identified candidates, we found thirteen solute carrier genes to be differentially regulated between young and aged animals (Fig. 5A). Among these, Slc13a5, mediating the cellular uptake of citrate is of special interest, as loss-of-function mutations are linked to seizures and epileptic encephalopathy [33]. Other solute carriers found to be upregulated in an age-dependent manner are Slc11a1 and Slc28a2, linked to iron uptake by macrophages or nucleoside transport, respectively.

Among the solute carriers found to be downregulated in aged mice (Fig. 5A), Slc16a9, which transports lactate, pyruvate and ketone bodies, is of note.

Next, we assessed age-dependent regulation of genes associated with endocytosis. Out of 351 genes associated with GO terms containing “endocytosis”, 20 genes were upregulated in aged meninges, while two genes were downregulated Fig. 5B). Of the upregulated genes, nine are linked to immunological processes, including in particular B cell specific *Ighm* (logFC 2.94), *Cd22* (logFC 2.24) and *Cd209d* (logFC 2.02). This data supports the increased presence of B cells in aged meninges, as also suggested by our analysis shown in Fig. 4.

### Aging influences meningeal protein secretion

In addition to cellular uptake, protein secretion by meningeal cells is important for CSF homeostasis by maintaining its proteome. Therefore, we evaluated the contribution of protein secretion by meningeal cells to CSF composition. Using the UniProt knowledge database, we assigned subcellular localization data to 12751 gene products expressed in meninges. Out of these 12751 genes, 1105 code for a protein annotated with cellular localization “secreted”. Interestingly, and supporting the notion that meningeal cells contribute to the CSF proteome, mean gene expression was about 3-times higher in the group of secreted proteins compared to the mean expression of all genes.

**Fig. 6:**
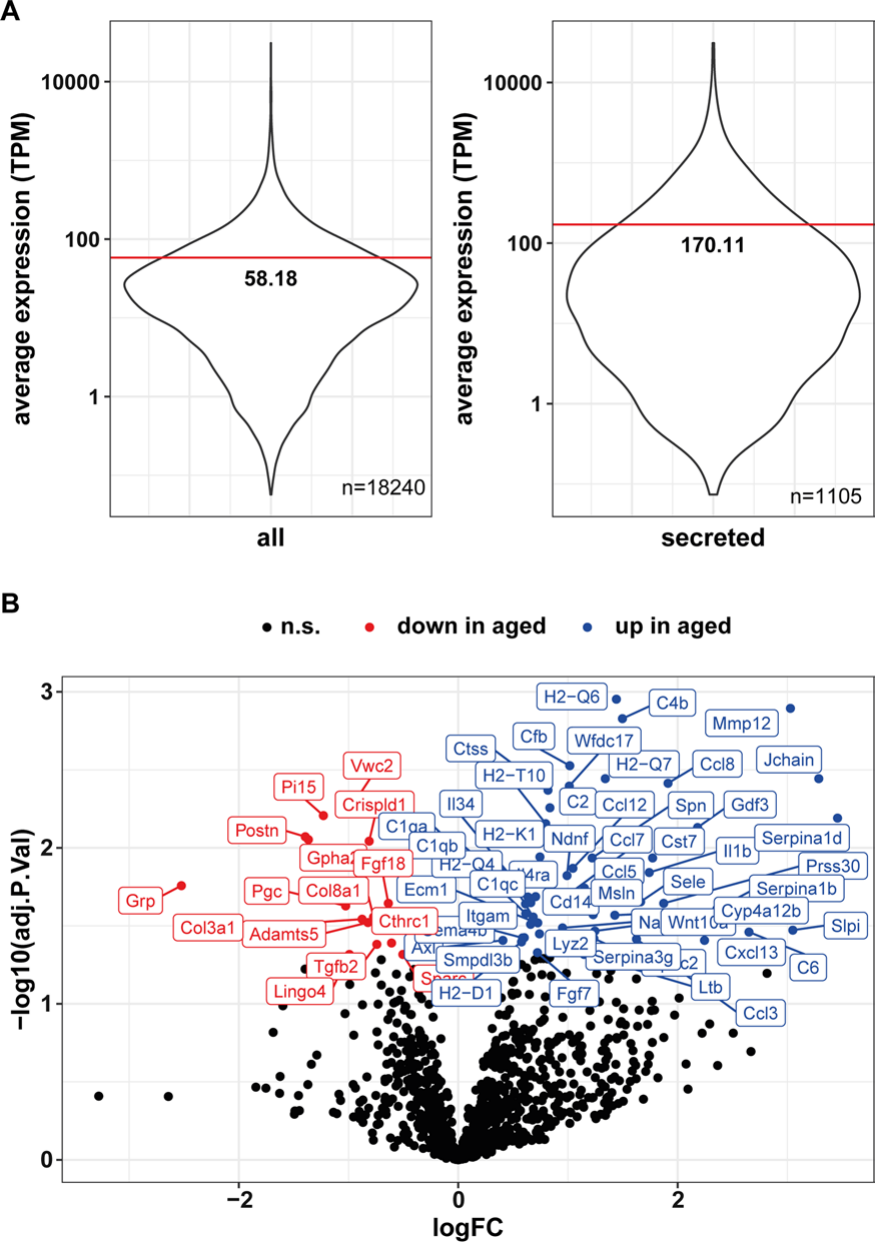
Aging alters the transcriptional profile of secreted proteins. **A** Based on average transcription of all samples irrespective of age, transcripts per million (TPM) were calculated and shown as violin plot for all identified genes (left panel) and for a list of genes coding for “secreted proteins” (right panel). The black line marks the mean gene expression in TPM. **B** A volcano plot highlighting differentially expressed genes from above gene list “secreted proteins”

**Fig. 7:**
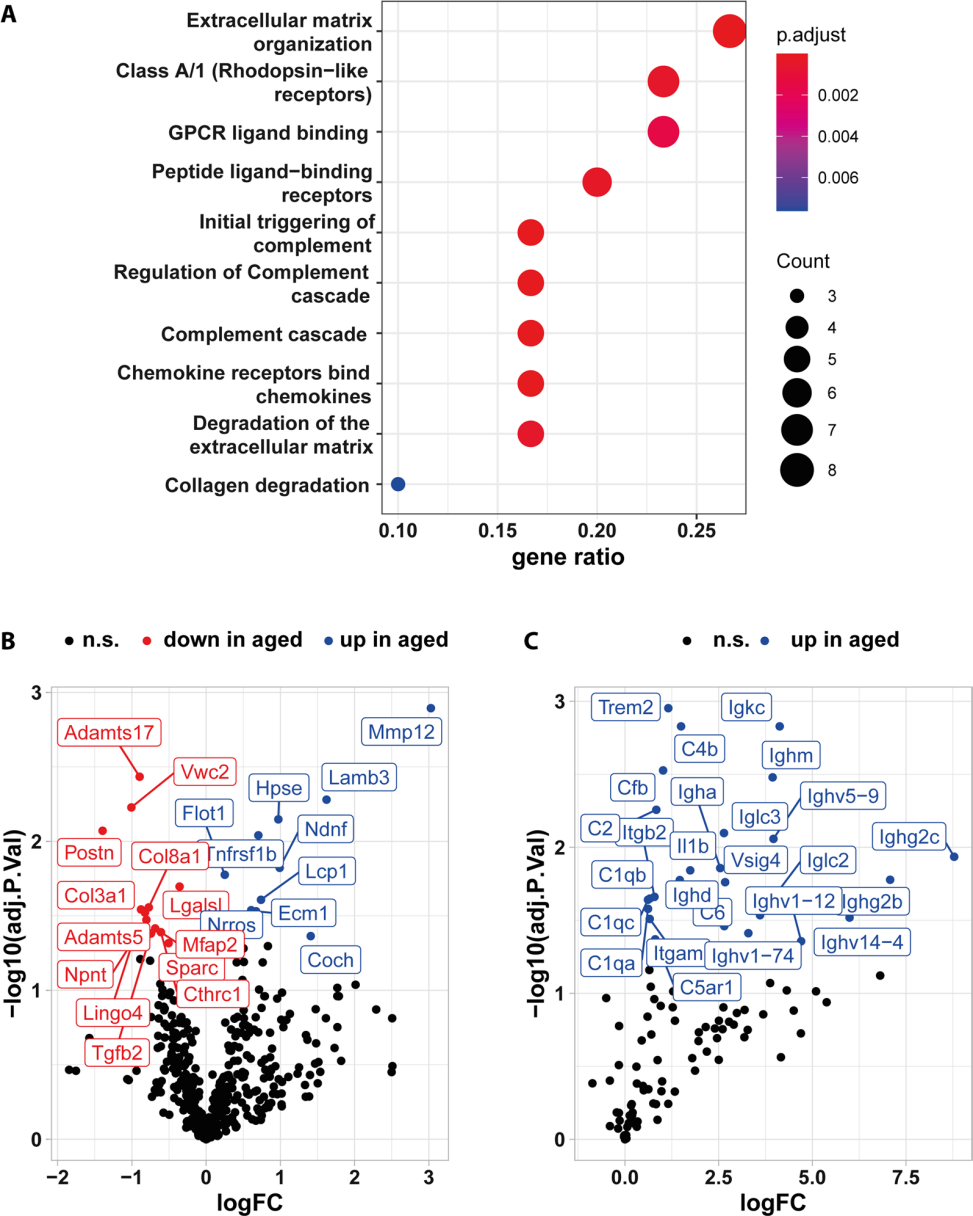
Reactome analysis of differentially regulated genes coding for secreted proteins. **A** Using the gene list “secreted proteins” from Fig. 6, reactome pathway analysis was performed using the Bioconductor ReactomePA package [55]. **B** A volcano plot of genes expressed in meninges and annotated with “extracellular matrix”. **C** A volcano plot of genes expressed in meninges and annotated with GO terms containing “complement”

Figure 6A secreted: 170.11 transcripts per million (TPM); all: 58.19 TPM). Among the genes coding for secreted proteins, 65 genes were differentially expressed with 50 genes upregulated in aged compared to young meninges (Fig. 6B). Using Reactome pathway analysis (Fig. 7A), affected pathways involving these differentially regulated genes coding for secreted proteins were centered on extracellular matrix (3/10), complement activation (3/10) and chemokine activity (1/10).

### Extracellular matrix and aging

As the meninges form the physical barrier surrounding and protecting the CNS, extracellular matrix composition is critical as it provides the structural basis for this function—reflected by the name dura (latin for hard, thick) for the outermost, collagenous layer of the meninges. Among the 542 genes associated with “extracellular matrix”, ten were upregulated while 13 were downregulated in aged compared to young meninges. Among these, genes coding for structural ECM components are affected with downregulation of collagen-coding genes *Col3a1* and *Col8a1*, suggesting an altered collagen composition of the extracellular matrix (Fig. 7B). In fact, we found 44 collagen genes expressed in meninges with levels averaged across all samples ranging from 1584 TPM for *Col1a* to 0.4 TPM for *Col6a6* (Additional file 1: Fig. S4A). Interestingly, expression of most collagen genes seemed to be affected by aging with borderline significance, further suggesting an impact on the collagenous ECM of aged meninges. With upregulation of *Lamb3* (coding for laminin β3), another structural component of ECM and in particular of the basal membrane is affected by aging. The impact of increased *Lamb3* expression, however, is unclear. The major laminin β-subunit, laminin β2, is more abundantly expressed in meninges by a factor of 250 (Additional file 1: Fig. S4B). However, as laminins are determining factors for cell invasion and migration, increased laminin β3 levels might contribute to the above described potential increase in B cell population in aged meninges. ECM composition is not only determined by structural proteins such as collagens, but also by ECM remodeling enzymes. The observed upregulation of *Mmp12* (coding for the matrix metallopeptidase Mmp12) suggests age-related changes to the ECM of meninges. In addition, Mmp12 is also known as macrophage elastase, indicating that the observed *Mmp12* upregulation might also reflect age-dependent changes to the meningeal macrophage population. Downregulation of *Adamsts5* and *Adamsts17* coding for proteases acting on proteoglycans and fibrillin, respectively, further supports age-dependent meningeal ECM remodeling. The upregulation of *Hpse* coding for heparanase active on proteoglycans, and *Mfap2* coding for Microfibril-Associated Protein 2, both connected to increased cell migration and invasion, might also link age-dependent ECM remodeling with changes to the meningeal immune cell population.

### Complement activation is impacted by aging

In addition to altered extracellular matrix, Reactome analysis suggested altered complement activation. Among 106 genes connected to complement activation, expression of 25 genes was increased in aged compared to young meninges (Fig. 7C). With *C1qa*, *C1qb*, *C1qc*, *C2*, *C4b*, *C6* and *Cfb*, seven core components of the classical and alternative complement system are upregulated. In addition to genes coding for complement components, the genes with the highest upregulation in this group are immune globulin genes linked to B cells and important for triggering complement activation.

### Alzheimer’s disease related changes to meningeal transcription

**Fig. 8:**
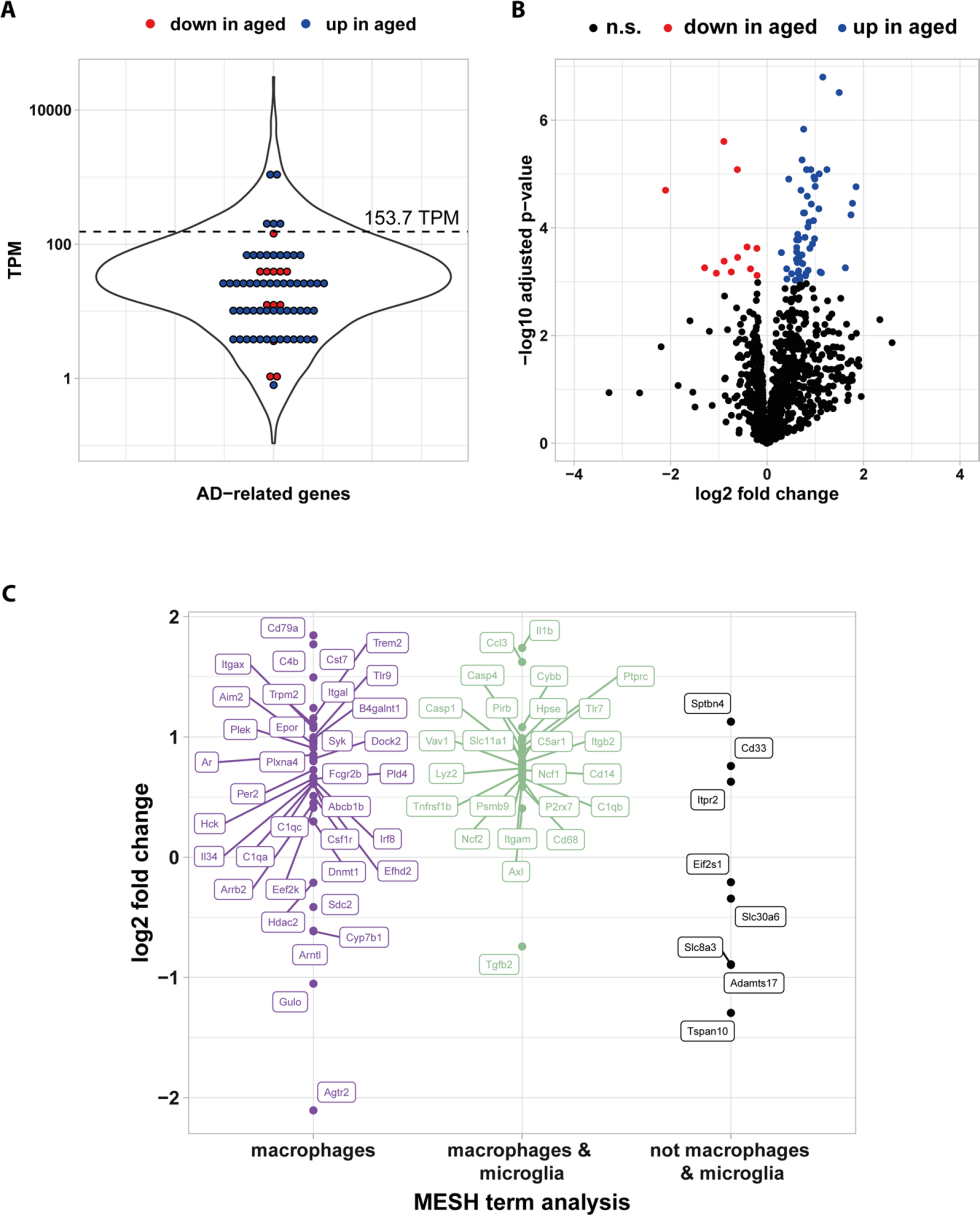
Age-dependent regulation of genes related to Alzheimer disease. **A** A list of 1237 genes detected in meninges and annotated with the MeSH term D000544/Alzheimer Disease was generated. The violin plot displays the distribution of expression level of these genes with gene expression level of differentially expressed genes individually marked. **B** A volcano plot of gene list from A. **C** Differentially expressed genes from gene list described in A were annotated using MeSH terms for D008264/macrophages and D017628/microglia and grouped. Shown is the log2 fold change for the three groups genes annotated with macrophages, macrophages and microglia and neither macrophages nor microglia

We previously reported on the neuroprotective function of MECs and their role in amyloid beta (Aβ) uptake linking these cells to Alzheimer’s disease (AD). Using Medical Subject Headings (MeSH) term analysis (Fig. 8), we analyzed our data set for age-related changes linked to AD. Based on this, we identified 1237 genes connected to AD. Of these genes, 12 were downregulated, while 57 genes were upregulated in aged compared to young animals (Fig. 8A, B). Among the highly regulated genes are *Cd79A* (logFC = 1.85) part of the B-cell antigen receptor complex, *Cst7* (logFC = 1.77), *Il1b* (logFC = 1.74), and *Trem2* (logFc = 1.16). Of particular interest are Cst7 and Trem2, known microglial markers associated with AD. Unlike the brain parenchyma, where microglia represent the predominant phagocytic cell type, the meninges are home to a specific subgroup of macrophages. We therefore used MeSH term analysis and found 61 out of 69 differentially regulated genes to be annotated with “microglia” and/or macrophages (Fig. 8C). This suggests an altered activation status of macrophages in the meninges during aging potentially connected to protective mechanisms involved in neurodegenerative processes.

## Discussion

Aging is accompanied by profound physiological changes. With its very limited regenerative capacity, the brain is especially affected by the effects of aging. The meninges, of minuscule volume compared to the large amount of neuronal and glial tissue of the CNS, are critical to maintaining brain function. In particular, the meninges are crucial to the immune-privileged status of the CNS and are clearly a first line of defense for the brain [36]. Connected to this, meninges are essential for maintaining CSF homeostasis through lymphatic drainage [37], but also through protein and small molecule secretion into as well as endocytotic uptake from the CSF [24].

To understand better the effect of aging on meningeal function, we compared meningeal transcriptional profiles between young and aged mice. We found prominent age-related changes in immunological landscape, structural integrity, and processes modulating CSF composition. Our data point to a clear shift towards a pro-inflammatory meningeal immune response as aging progresses. We find clear indication for increased numbers of T and B lymphocytes in the meninges of aged mice. This is in line with the single cell-resolution immune cell atlas of the dural sinus of young and aged mice [38]. There, aging was accompanied by increased numbers of CD4 and CD8 T cells. Furthermore, our observations of increased T and B lymphocytes are also in line with reports showing an age-dependent increase in meningeal T cells and in particular Foxp3^+^ T_reg_ while at the same time levels of T cells positive for the chemokine receptor Ccr7 were decreased [15, 19]. Interestingly, diminished *Ccr7* expression was linked to an observed increase of the T_reg_ population, and exacerbated disease phenotype in 5xFAD Alzheimer mice [15]. In our data set, neither *Ccr7* nor *Foxp3* expression were found to be significantly altered. However, we found the several genes encoding chemokines connected to T cell attraction and homing including *Ccl3*, *Ccl5*, *Ccl7*, and *Ccl8* [39] to be upregulated in aged animals. These findings support an age-dependent impact on T cell homing and retention in the meninges.

Similar to T cells, we found increased expression of B cell-related pathways in line with Brioschi et al. [17], who recently discovered the meningeal B cell population to originate from the calvaria bone marrow, consistent with a specialized B cell subset tuned to the needs of the CNS. During aging, peripheral B cells and plasma cells were shown to invade the meninges potentially threatening the CNS immune privilege. In our data set, we indeed detected expression of plasma cell markers *Cxcr4*, *Cd93*, *Il6R* and *Cd44* [40] supporting the presence of plasma cells in aged meninges.

While our analysis supports altered meningeal populations of T and B lymphocytes, our data does not hint towards a marked increase in meningeal macrophages. Consistent with this, we do not find increased expression of the meningeal macrophage marker *Mrc1/Cd206* [41]. However, in IPA a clear trend towards altered activity of these cells is apparent. Interestingly, genes with age-dependent regulation related to AD can mostly be attributed to macrophages and microglia. In this context, the highly significant increase of *Trem2* expression during aging is of special interest. Trem2 is a transmembrane receptor found on cells of myeloid origin. Rare partial loss-of-function variants of *Trem2* are linked to increased risk to develop AD. Engagement of Trem2 induces a signaling cascade leading to increased cellular activity via Syk and PI3 kinase [42]—also upregulated in aged meninges. Thus, our data supports a heightened state of activity for meningeal macrophages in response to aging. In line with this, we also find upregulation of genes related to endocytosis/phagocytosis, further supporting increased toxic metabolite clearance with age. Similarly, the observed upregulation of complement components in aged meninges might be connected to increased macrophage activity. Complement activation is linked to various age-related neurodegenerative disorders and is known to regulate microglial activation [43]. Among others, the complement receptor *Vsig4* is upregulated in aged meninges about sixfold compared to young meninges. As Vsig4 is mainly found on monocytes/macrophages, this further supports a more active meningeal macrophage population in aged mice.

The complex meningeal architecture suggests an equally complex meningeal immune system. Indeed, meningeal immune cells show distinct sub-meningeal distribution. For example, barrier macrophages are mainly found in high densities in the dura and the pia mater, but less so in the subarachnoid space [44]. Similarly, γδ-T cells are more prevalent in the dura than the arachnoid [16]. While our data set provides insight into potential age-dependent changes to the meningeal immune system, it remains unclear whether these changes affect all meningeal layers or are limited to individual meningeal layers. To answer these questions, single cell sequencing with sub-meningeal resolution would be necessary.

Meningeal extracellular matrix remodeling seems to be a consequence of aging. We found differences in the expression of various ECM components including laminins and collagens, but also proteases responsible for ECM proteolysis. An altered ECM composition would not only impact the mechanical properties of the meninges, but could potentially contribute to differences in immune cell populations associated with age. Also, the observed eightfold increased expression of *Mmp12,* also known as macrophage metalloelastase, likely reflects age-related changes to the meningeal macrophage activity status. In addition, decreases in collagen expression points to a MEC-driven ECM remodeling. Based on the high expression level of various collagens, only MECs can conceivably produce these large amounts of ECM. Whether this attenuation of collagen expression extends also to other MEC functions remains unclear. In fact, we did not find indications for a substantial loss of secretory capacity of these cells. When looking at the top ten genes coding for secreted proteins including *Apoe, B2m, Chgb, Cst3, Ctsd, Gpx3, Igfbp5, Mgp, Enpp2,* and *Ttr*—responsible for 6.5% of total transcription—we did not find significant differences between young and aged mice.

Our analysis of solute carrier gene expression revealed among others differential expression of *Slc13a5* and *Slc16a9* involved in the transport of citrate or lactate, pyruvate and ketone bodies, respectively. These metabolites are critical for energy metabolism and differential expression of their transporters might be linked to age-related changes metabolomic changes in the CSF [45–47]. Produced by astrocytes, citrate is released into the CSF at a concentration of up to 0.4 mM [34] and seems to be involved in the chelation of divalent ions such as Ca^2+^ and Zn^2+^. Slc13a5 is significantly upregulated in aged compared to young mice (by 1.8 fold) suggesting increased citrate clearance from the CSF by meningeal cells. In addition, lactate is important in neuronal energy metabolism [35]. At the level of the astrocyte–neuron lactate shuttle, astrocytes release lactate to surrounding neurons, enabling efficient neuronal ATP generation. However, whether MECs or other meninges-resident cells are directly involved in contributing lactate for neuronal consumption is unknown.

We were able to identify several regulatory pathways affected by aging. And in particular our findings regarding age-related changes to meningeal immune responses corroborate previous reports [15, 16, 38]. However, we also want to point out that bulk RNASeq is not able to discriminate between different cell types present in the analyzed tissue or prove altered function of these cells. And although the main cellular component of our analyzed samples are MECs, we cannot rule out that certain observations attributed here to changes in MEC function are potentially confounded by the presence of other cell types in our samples. Also, it remains unclear which MEC subtype undergoes these age-dependent changes as our sample contains based on marker expression not only dural, but also arachnoid and likely to some extent pial meningeal fibroblasts (Additional file 1: Fig. S2). Another limitation of our study is the reliance on transcriptional profiling. While gene expression studies paired with knowledge-based bioinformatics analyses can point towards affected cellular pathways such studies cannot provide functional insight. Additional experimentation would be necessary to draw functional conclusions about age-related changes to the meninges.

In our study, we compared the transcriptional profile of young and aged meninges. Our results indicate that the meninges play an important role in the immunological defense of the CNS. In particular, we find evidence for an age-related switch to a pro-inflammatory meningeal phenotype that involves the adaptive as well as the innate immune system. As immunological processes in the brain are intimately linked to the maintenance of cognitive function and as mis-directed immune responses are connected to neurodegenerative processes, our results highlight the importance of the oftentimes overlooked meninges for CNS function.

## Materials and methods

### Animal experimentation

Animal experiments were approved by the Cantonal Veterinary Office of Basel

Stadt (licence 3023). C57BL/6JRj mice were bred and kept under specific pathogen-free conditions. Young mice were between 9 and 15 weeks, aged mice were between 80 and 97 weeks at the time of the experiment. All mice used in this experiment were female.

### Isolation of meninges

Meninges were isolated according to Louveau et. al [31]. In short, mice were anesthetized with 100 mg/kg ketamine and 20 mg/kg xylazine in 0.9% saline. After reaching deep anesthesia, animals were transcardially perfused with PBS/heparin (5 U/ml, Sigma-Aldrich H3393) using a syringe pump (PHD Ultra, Harvard Apparatus) at a rate of 2 ml/min for at least 5 min until the liver showed clear signs of exsanguination. Next, the head was severed above the shoulders, skin and flesh were removed from the skull and the optic nerves were severed. After removing the lower jaw and the nasal bone, the skull was opened and the brain was carefully removed. Meningeal tissue was dissected from the skull bone using forceps; no material was obtained by dissecting mengines from the brain itself. Meninges were immediately transferred into buffer RLT buffer for RNA isolation (see below).

### RNA isolation and NGS sequencing

RNA was isolated using RNeasy Micro Kit (Qiagen, 74004). Isolated total RNA was processed and sequenced at Novogen (Oxford) using their low input protocol to obtain paired sequences of 300 bp.

### Bioinformatic analysis

Sequences were aligned using subread v2.0.0 [48] against a full-index of the GRCm38 (mm10) mouse reference genome. Features were counted using featureCounts v2.0.0 against the built-in annotations [49]. The compute platform was a four node Raspberry Pi 4B 8 GB cluster running slurm 19.05.5 scheduler under aarch64 Ubuntu 20.04 LTS.

Differential gene expression was assessed using R 4.1.0 and the Bioconductor packages limma [50] and edgeR [51]. Following feature counting, transcripts with less than 10 counts were filtered as described [52]. Pathway analysis was performed using Ingenuity (Qiagen [53]) and the Bioconductor [54] package ReactomePA [55]. GO and MeSH term analysis were performed using Bioconductor packages biomaRt [56] and MeSH.Mmu.eg.db [57], respectively. Plotting was done using R packages ggplot2 [58] and circlize [59]. To account for multiple testing, p-value adjustment was performed according to Benjamini-Hochberg.

## Supplementary Information

**Additional file 1: Figure S1.** Processing of RNASeq data. **Figure S2.** Comparing expression of known meningeal markers between young and aged animals. **Figure S3**. Cluster analysis of IPA. **Figure S4.** Overview expression of collagens and laminins. **Table S1.** Sample overview.

## Data Availability

Data is available at https://www.ncbi.nlm.nih.gov/geo/.

## References

[1] Bifari F, Decimo I, Pino A, Llorens-Bobadilla E, Zhao S, Lange C (2017). Neurogenic radial glia-like cells in meninges migrate and differentiate into functionally integrated neurons in the neonatal cortex. Cell Stem Cell.

[2] Reiss K, Mentlein R, Sievers J, Hartmann D (2002). Stromal cell-derived factor 1 is secreted by meningeal cells and acts as chemotactic factor on neuronal stem cells of the cerebellar external granular layer. Neuroscience.

[3] Choe Y, Siegenthaler JA, Pleasure SJ (2012). A cascade of morphogenic signaling initiated by the meninges controls corpus callosum formation. Neuron.

[4] Hu X, Deng Q, Ma L, Li Q, Chen Y, Liao Y (2020). Meningeal lymphatic vessels regulate brain tumor drainage and immunity. Cell Res.

[5] Louveau A, Plog BA, Antila S, Alitalo K, Nedergaard M, Kipnis J (2017). Understanding the functions and relationships of the glymphatic system and meningeal lymphatics. J Clin Invest.

[6] Decimo I, Dolci S, Panuccio G, Riva M, Fumagalli G, Bifari F (2021). Meninges: a Widespread niche of neural progenitors for the brain. Neuroscientist.

[7] Nakada T, Kwee IL (2019). Fluid dynamics inside the brain barrier: current concept of interstitial flow, glymphatic flow, and cerebrospinal fluid circulation in the brain. Neuroscientist.

[8] Killer HE, Laeng HR, Groscurth P (1999). Lymphatic capillaries in the meninges of the human optic nerve. J Neuroophthalmol.

[9] Da Mesquita S, Fu Z, Kipnis J (2018). The meningeal lymphatic system: a new player in neurophysiology. Neuron.

[10] Da Mesquita S, Louveau A, Vaccari A, Smirnov I, Cornelison RC, Kingsmore KM (2018). Functional aspects of meningeal lymphatics in ageing and Alzheimer's disease. Nature.

[11] Zou W, Pu T, Feng W, Lu M, Zheng Y, Du R (2019). Blocking meningeal lymphatic drainage aggravates Parkinson's disease-like pathology in mice overexpressing mutated alpha-synuclein. Transl Neurodegener.

[12] Nayak D, Zinselmeyer BH, Corps KN, McGavern DB (2012). In vivo dynamics of innate immune sentinels in the CNS. Intravital.

[13] O'Brien CA, Overall C, Konradt C, O'Hara Hall AC, Hayes NW, Wagage S (2017). CD11c-expressing cells affect regulatory t cell behavior in the meninges during central nervous system infection. J Immunol.

[14] Ribeiro M, Brigas HC, Temido-Ferreira M, Pousinha PA, Regen T, Santa C (2019). Meningeal gammadelta T cell-derived IL-17 controls synaptic plasticity and short-term memory. Sci Immunol.

[15] Da Mesquita S, Herz J, Wall M, Dykstra T, de Lima KA, Norris GT (2021). Aging-associated deficit in CCR7 is linked to worsened glymphatic function, cognition, neuroinflammation, and beta-amyloid pathology. Sci Adv.

[16] Alves de Lima K, Rustenhoven J, Da Mesquita S, Wall M, Salvador AF, Smirnov I (2020). Meningeal gammadelta T cells regulate anxiety-like behavior via IL-17a signaling in neurons. Nat Immunol.

[17] Brioschi S, Wang WL, Peng V, Wang M, Shchukina I, Greenberg ZJ (2021). Heterogeneity of meningeal B cells reveals a lymphopoietic niche at the CNS borders. Science.

[18] Goldmann T, Wieghofer P, Jordao MJ, Prutek F, Hagemeyer N, Frenzel K (2016). Origin, fate and dynamics of macrophages at central nervous system interfaces. Nat Immunol.

[19] Mrdjen D, Pavlovic A, Hartmann FJ, Schreiner B, Utz SG, Leung BP (2018). High-dimensional single-cell mapping of central nervous system immune cells reveals distinct myeloid subsets in health, aging, and disease. Immunity.

[20] Derk J, Jones HE, Como C, Pawlikowski B, Siegenthaler JA (2021). Living on the edge of the CNS: meninges cell diversity in health and disease. Front Cell Neurosci.

[21] DeSisto J, O'Rourke R, Jones HE, Pawlikowski B, Malek AD, Bonney S (2020). Single-cell transcriptomic analyses of the developing meninges reveal meningeal fibroblast diversity and function. Dev Cell.

[22] Dorrier CE, Jones HE, Pintaric L, Siegenthaler JA, Daneman R (2022). Emerging roles for CNS fibroblasts in health, injury and disease. Nat Rev Neurosci.

[23] Zeleny TNC, Kohler C, Neutzner A, Killer HE, Meyer P (2017). Cell-cell interaction proteins (gap junctions, tight junctions, and desmosomes) and water transporter aquaporin 4 in meningothelial cells of the human optic nerve. Front Neurol.

[24] Hemion C, Li J, Kohler C, Scholl HPN, Meyer P, Killer HE (2020). Clearance of neurotoxic peptides and proteins by meningothelial cells. Exp Cell Res.

[25] Li J, Fang L, Killer HE, Flammer J, Meyer P, Neutzner A (2013). Meningothelial cells as part of the central nervous system host defence. Biol Cell.

[26] Li J, Fang L, Meyer P, Killer HE, Flammer J, Neutzner A (2014). Anti-inflammatory response following uptake of apoptotic bodies by meningothelial cells. J Neuroinflammation.

[27] Pikor NB, Cupovic J, Onder L, Gommerman JL, Ludewig B (2017). Stromal cell niches in the inflamed central nervous system. J Immunol.

[28] Wu Z, Zhang J, Nakanishi H (2005). Leptomeningeal cells activate microglia and astrocytes to induce IL-10 production by releasing pro-inflammatory cytokines during systemic inflammation. J Neuroimmunol.

[29] Fan B, Bordigari G, Flammer J, Killer HE, Meyer P, Neutzner A (2012). Meningothelial cells participate in immunological processes in the cerebrospinal fluid. J Neuroimmunol.

[30] Hao J, Kohler C, van den Dorpel H, Scholl HPN, Meyer P, Killer HE (2020). The extracellular matrix composition of the optic nerve subarachnoid space. Exp Eye Res.

[31] Louveau A, Filiano AJ, Kipnis J (2018). Meningeal whole mount preparation and characterization of neural cells by flow cytometry. Curr Protoc Immunol.

[32] Law CW, Chen Y, Shi W, Smyth GK (2014). voom: precision weights unlock linear model analysis tools for RNA-seq read counts. Genome Biol.

[33] Thevenon J, Milh M, Feillet F, St-Onge J, Duffourd Y, Juge C (2014). Mutations in SLC13A5 cause autosomal-recessive epileptic encephalopathy with seizure onset in the first days of life. Am J Hum Genet.

[34] Westergaard N, Waagepetersen HS, Belhage B, Schousboe A (2017). Citrate, a ubiquitous key metabolite with regulatory function in the CNS. Neurochem Res.

[35] Brooks GA (2018). The science and translation of lactate shuttle theory. Cell Metab.

[36] Engelhardt B, Coisne C (2011). Fluids and barriers of the CNS establish immune privilege by confining immune surveillance to a two-walled castle moat surrounding the CNS castle. Fluids Barriers CNS.

[37] Meyer C, Martin-Blondel G, Liblau RS (2017). Endothelial cells and lymphatics at the interface between the immune and central nervous systems: implications for multiple sclerosis. Curr Opin Neurol.

[38] Rustenhoven J, Drieu A, Mamuladze T, de Lima KA, Dykstra T, Wall M (2021). Functional characterization of the dural sinuses as a neuroimmune interface. Cell.

[39] Strazza M, Mor A (2017). Consider the chemokines: a review of the interplay between chemokines and T cell subset function. Discov Med.

[40] Brynjolfsson SF, Persson Berg L, Olsen Ekerhult T, Rimkute I, Wick MJ, Martensson IL (2018). Long-lived plasma cells in mice and men. Front Immunol.

[41] Mammana S, Fagone P, Cavalli E, Basile MS, Petralia MC, Nicoletti F (2018). The role of macrophages in neuroinflammatory and neurodegenerative pathways of Alzheimer's disease, amyotrophic lateral sclerosis, and multiple sclerosis: pathogenetic cellular effectors and potential therapeutic targets. Int J Mol Sci.

[42] Carmona S, Zahs K, Wu E, Dakin K, Bras J, Guerreiro R (2018). The role of TREM2 in Alzheimer's disease and other neurodegenerative disorders. Lancet Neurol.

[43] Fatoba O, Itokazu T, Yamashita T (2022). Complement cascade functions during brain development and neurodegeneration. FEBS J.

[44] Rua R, McGavern DB (2018). Advances in meningeal immunity. Trends Mol Med.

[45] Zebhauser PT, Berthele A, Goldhardt O, Diehl-Schmid J, Priller J, Ortner M (2022). Cerebrospinal fluid lactate levels along the Alzheimer's disease continuum and associations with blood-brain barrier integrity, age, cognition, and biomarkers. Alzheimers Res Ther.

[46] Peters K, Herman S, Khoonsari PE, Burman J, Neumann S, Kultima K (2021). Metabolic drift in the aging nervous system is reflected in human cerebrospinal fluid. Sci Rep.

[47] Panyard DJ, Yu B, Snyder MP (2022). The metabolomics of human aging: Advances, challenges, and opportunities. Sci Adv.

[48] Liao Y, Smyth GK, Shi W (2013). The Subread aligner: fast, accurate and scalable read mapping by seed-and-vote. Nucleic Acids Res.

[49] Liao Y, Smyth GK, Shi W (2014). featureCounts: an efficient general purpose program for assigning sequence reads to genomic features. Bioinformatics.

[50] Ritchie ME, Phipson B, Wu D, Hu Y, Law CW, Shi W (2015). limma powers differential expression analyses for RNA-sequencing and microarray studies. Nucleic Acids Res.

[51] Robinson MD, McCarthy DJ, Smyth GK (2010). edgeR: a Bioconductor package for differential expression analysis of digital gene expression data. Bioinformatics.

[52] Law CW, Alhamdoosh M, Su S, Dong X, Tian L, Smyth GK (2016). RNA-seq analysis is easy as 1-2-3 with limma, Glimma and edgeR. F1000Res.

[53] Kramer A, Green J, Pollard J, Tugendreich S (2014). Causal analysis approaches in ingenuity pathway analysis. Bioinformatics.

[54] Huber W, Carey VJ, Gentleman R, Anders S, Carlson M, Carvalho BS (2015). Orchestrating high-throughput genomic analysis with Bioconductor. Nat Methods.

[55] Yu G, He QY (2016). ReactomePA: an R/Bioconductor package for reactome pathway analysis and visualization. Mol Biosyst.

[56] Durinck S, Spellman PT, Birney E, Huber W (2009). Mapping identifiers for the integration of genomic datasets with the R/Bioconductor package biomaRt. Nat Protoc.

[57] Tsuyuzaki K, Morota G, Ishii M, Nakazato T, Miyazaki S, Nikaido I (2015). MeSH ORA framework: R/Bioconductor packages to support MeSH over-representation analysis. BMC Bioinformatics.

[58] Hadley W (2016). ggplot2: elegant graphics for data analysis.

[59] Gu Z, Gu L, Eils R, Schlesner M, Brors B (2014). circlize implements and enhances circular visualization in R. Bioinformatics.

