# Supplementary material for: Impact of aging on meningeal gene expression
Source: Fluids Barriers CNS. 2023 Feb 6;20:12. doi: 10.1186/s12987-023-00412-9 (PMC9903605; doi:10.1186/s12987-023-00412-9)
Supplement: Supplementary file 1 — Additional file 1: Figure S1. Processing of RNASeq data. Figure S2. Comparing expression of known meningeal markers between young and aged animals. Figure S3. Cluster analysis of IPA. Figure S4. Overview expression of collagens and laminins. Table S1. Sample overview. [file 12987_2023_412_MOESM1_ESM.pdf]

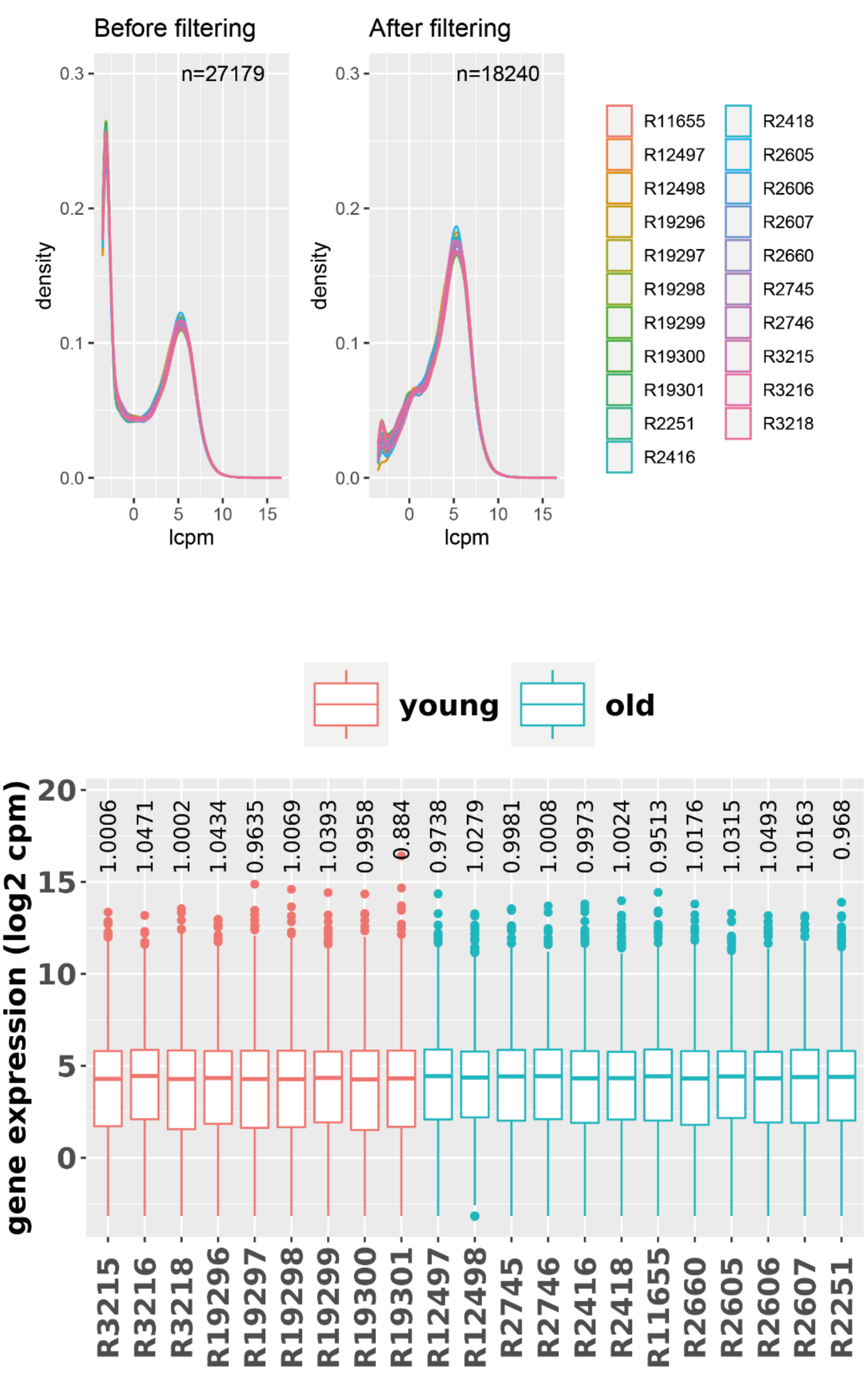

Figure S1 Processing of RNASeq data.

(A) Log2 counts per million (lcpm) were calculated for raw count data before (left panel) or after removal (right panel) of low expressing genes using a < 1 counts per million (cpm) threshold. Shown are density plots of lcpm values for all samples included in this study. (B) Using edgeR, normalization factors to account for different library sizes were calculated. The box plot shows normalized gene expression in lcpm for each sample with the associated normalization factor.

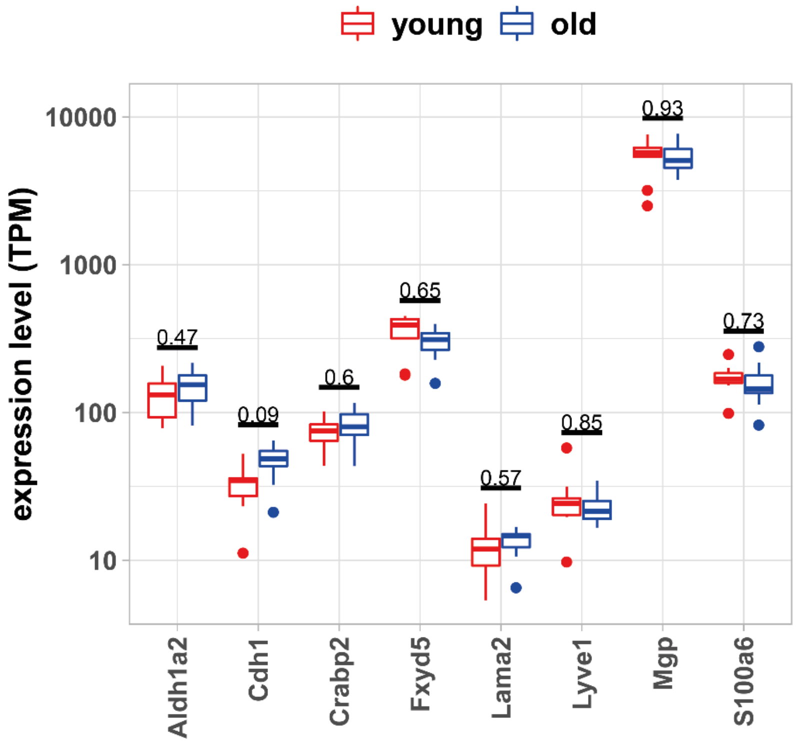

Figure S2:

Comparing expression of known meningeal markers between young and aged animals. P-values shown were adjusted for multiple testing according to Benjamini-Hochberg.

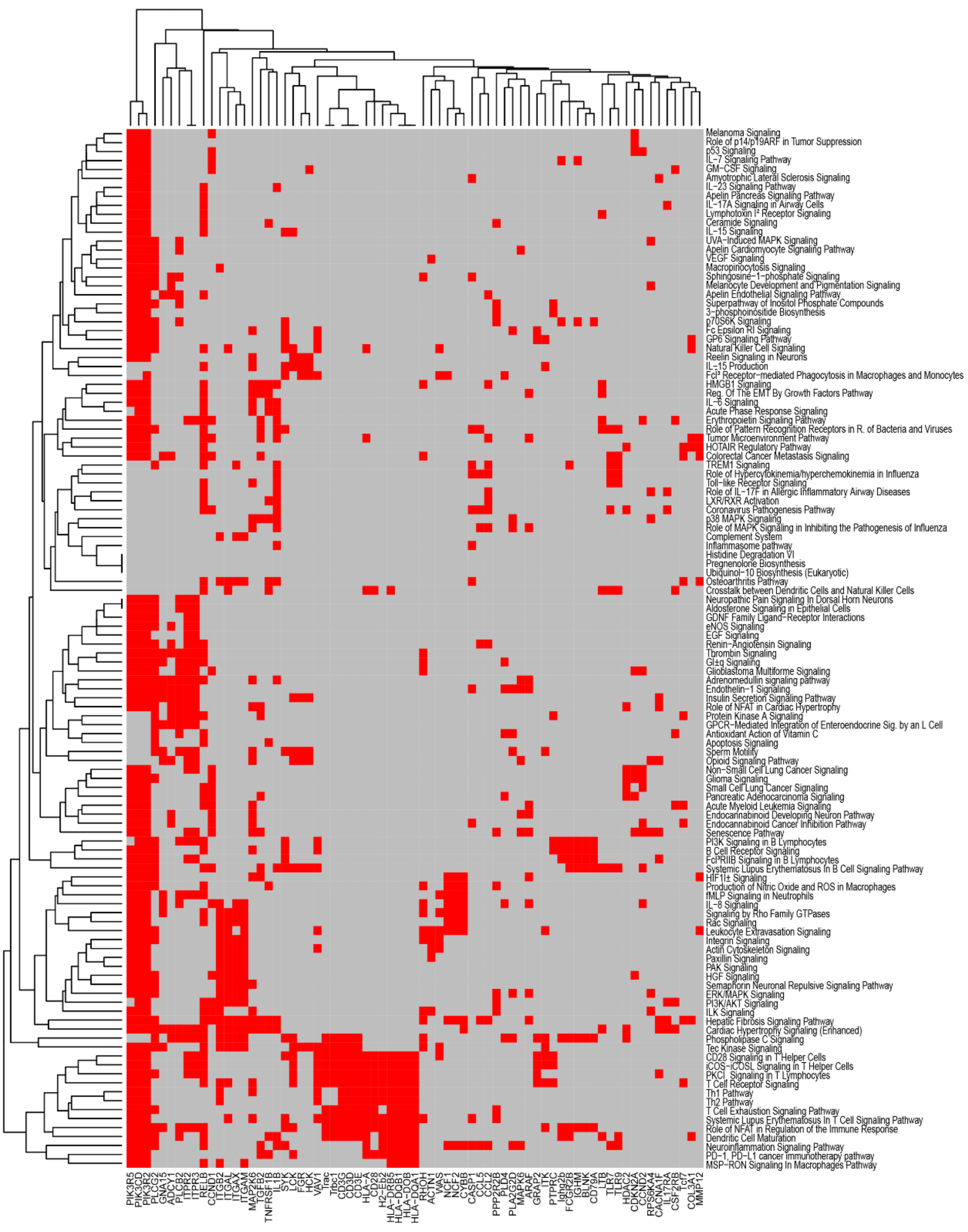

Figure S3 Cluster analysis of IPA

(A) Pathways identified by IPA to be affected by aging and their associated genes were clustered using Euclidean distance as implemented in the heatmap.2 function of the R package gplots (1). Only genes with more than five occurrences among the 116 identified pathways were included in the analysis.

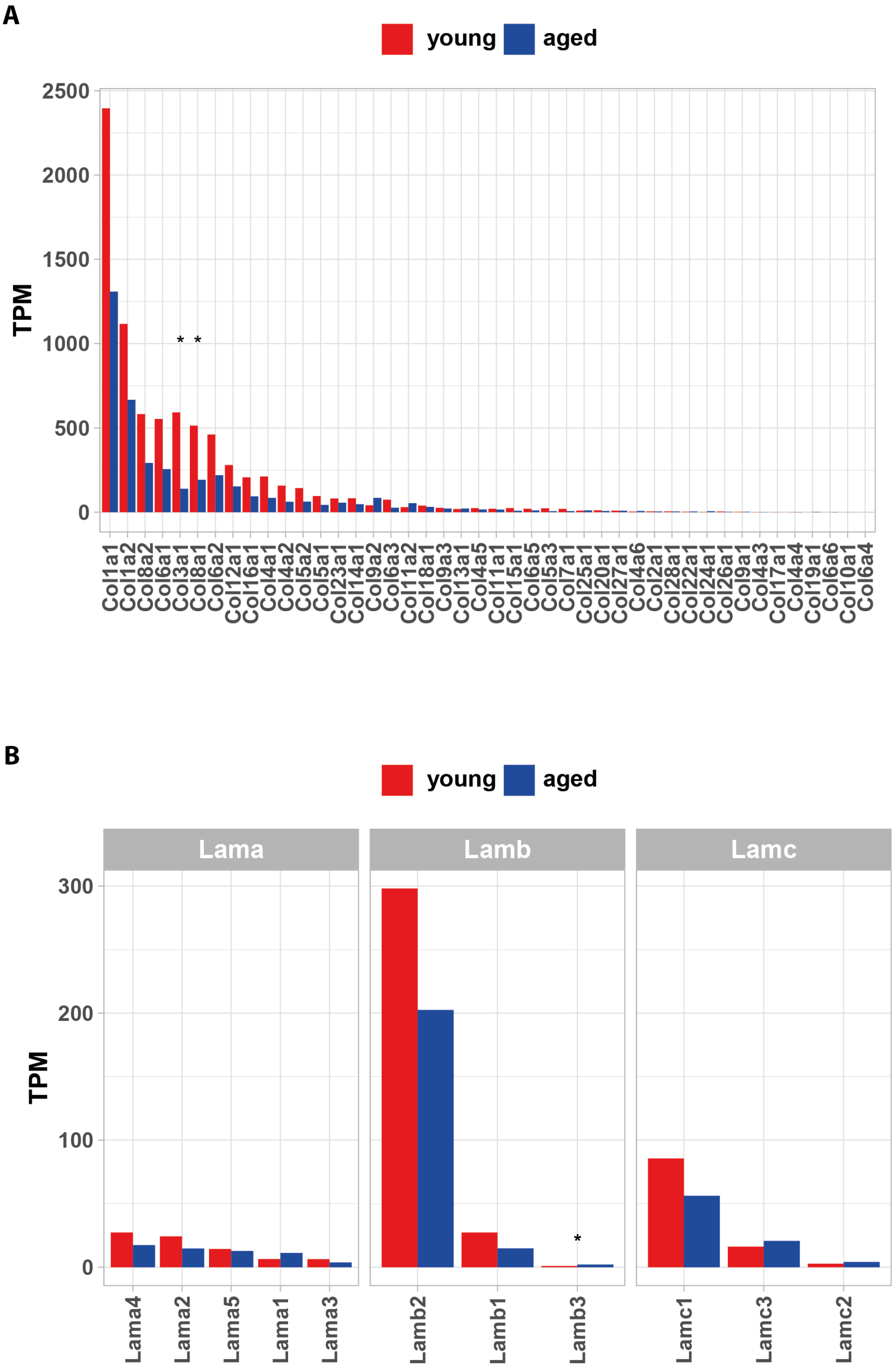

Figure S4 Overview expression of collagens and laminins

(A) A list of 44 genes coding for collagens and expressed in meninges was assembled. The bar graph represents expression of these genes in TPM between young and aged meninges. * - adjusted p value < 0.05. **(B)** A list of eleven genes coding for α, β, and γ laminin subunits and expressed in meninges was generated. The bar graph displays absolute gene expression in TPM. * - adjusted p value < 0.05.

Sample	Age (weeks)	Total RNA (ug)	RIN	# of raw sequences	# of identified features
R2605	92	0.324	8.4	38933306	15729936
R2606	92	0.182	8.7	40732244	16230153
R2607	92	0.144	4.1	46446674	18895771
R2745	85	0.144	9.2	41137706	16770167
R2660	90	0.147	3.7	53149366	21888333
R2746	85	0.232	9.1	42037828	17143120
R19296	15	0.24	9	51211418	20834215
R19297	15	0.13	8.9	59645404	24224799
R19298	15	0.18	3.9	40279466	16233067
R19299	15	0.112	4	42799668	16870469
R19300	15	0.16	7.9	43949494	17847377
R19301	15	0.088	3.9	44147192	18162963
R2251	97	0.04563	5.60	45923162	18570606
R2418	86	0.02975	6.20	40375652	16165252
R3216	10	0.05318	5.70	41463118	16218832
R12498	80	0.03776	6.20	41589084	16845699
R11655	89	0.06036	5.30	52976638	21134683
R3215	9	0.07178	5.60	38205860	15095769
R12497	80	0.03701	6.20	47502128	18785257
R2416	86	0.03983	5.90	43873890	17699940
R3218	86	0.05605	6.30	40058188	15879067

Table S1 Sample overview. Shown are unique animal identifiers, age of the animal at the time of sacrifice, total amount of RNA obtained from isolated meninges measured by NanoDrop (ThermoFisher Scientific), the RNA integrity value (RIN) obtained using BioAnalyzer (Agilent), the number of raw sequences obtained from sequencing, and the number of sequences assigned to a genomic feature.

Supplemental file description

File S1 Comma-separated file containing 18240 rows of data for genes identified in meninges isolated from mice. The file contains the columns: NCBI gene ID => NCBI gene id (https://www.ncbi.nlm.nih.gov/gene); ENSEMBL => ENSEMBL gene id (ensembl.org) ;
logFC => log2 foldchange in gene expression between young and old mice; adj.P.Val => p-value adjusted for multiple testing; AvgCountOld => average feature count across samples from young animals; AvgCountYoung => average feature count across samples from young animals; AvgCount => average feature count across all samples; SYMBOL => gene symbol (https://www.ncbi.nlm.nih.gov/gene); meanTPMYoung => average expression level in young animals expressed as TPM; meanTPMOld => average expression level in old animals expressed as TPM;

Supplemental References

1. Warnes G, Bolker B, Bonebakker L, Gentleman R, Huber W, Liaw A, et al. gplots: Various R Programming Tools for Plotting Data. 3.1.3 ed2022.
